# Synergistic effect of chitosan-alginate composite hydrogel enriched with ascorbic acid and alpha-tocopherol under hypoxic conditions on the behavior of mesenchymal stem cells for wound healing

**DOI:** 10.1186/s13287-023-03567-2

**Published:** 2023-11-13

**Authors:** Maryam Ghahremani-nasab, Naeimeh Akbari‑Gharalari, Azizeh Rahmani Del Bakhshayesh, Armita Ghotaslou, Abbas Ebrahimi-kalan, Mahdi Mahdipour, Ahmad Mehdipour

**Affiliations:** 1 Department of Tissue Engineering, Faculty of Advanced Medical Sciences, Tabriz University of Medical Sciences, Tabriz, Iran; 2 Department of Neurosciences and Cognition, Faculty of Advanced Medical Sciences, Tabriz University of Medical Sciences, Tabriz, Iran; 3 Department of Medical Biotechnology, Faculty of Advanced Medical Sciences, Tabriz University of Medical Sciences, Tabriz, Iran; 4 Stem Cell Research Centre, Tabriz University of Medical Sciences, Tabriz, Iran

**Keywords:** Wound healing, Regenerative medicine, Hydrogel, Ascorbic acid, α-Tocopherol

## Abstract

**Background:**

In regenerative medicine, especially skin tissue engineering, the focus is on enhancing the quality of wound healing. Also, several constructs with different regeneration potentials have been used for skin tissue engineering. In this study, the regenerative properties of chitosan-alginate composite hydrogels in skin wound healing under normoxic and hypoxic conditions were investigated in vitro.

**Methods:**

The ionic gelation method was used to prepare chitosan/alginate (CA) hydrogel containing CA microparticles and bioactive agents [ascorbic acid (AA) and α-tocopherol (TP)]. After preparing composite hydrogels loaded with AA and TP, the physicochemical properties such as porosity, pore size, swelling, weight loss, wettability, drug release, and functional groups were analyzed. Also, the hemo-biocompatibility of composite hydrogels was evaluated by a hemolysis test. Then, the rat bone marrow mesenchymal stem cells (rMSCs) were seeded onto the hydrogels after characterization by flow cytometry. The survival rate was analyzed using MTT assay test. The hydrogels were also investigated by DAPI and H&E staining to monitor cell proliferation and viability. To induce hypoxia, the cells were exposed to CoCl_2_. To evaluate the regenerative potential of rMSCs cultured on CA/AA/TP hydrogels under hypoxic conditions, the expression of the main genes involved in the healing of skin wounds, including HIF-1α, VEGF-A, and TGF-β1, was investigated by real-time PCR.

**Results:**

The results demonstrated that the prepared composite hydrogels were highly porous, with interconnected pores that ranged in sizes from 20 to 188 μm. The evaluation of weight loss showed that the prepared hydrogels have the ability to biodegrade according to the goals of wound healing. The reduction percentage of CA/AA/TP mass in 21 days was reported as 21.09 ± 0.52%. Also, based on wettability and hemolysis tests of the CA/AA/TP, hydrophilicity (*θ* = 55.6° and 53.7°) and hemocompatibility with a hemolysis ratio of 1.36 ± 0.19 were evident for them. Besides, MTT assay, DAPI, and H&E staining also showed that the prepared hydrogels provide a suitable substrate for cell growth and proliferation. Finally, based on real-time PCR, increased expression levels of VEGF and TGF-β1 were observed in rMSCs in hypoxic conditions cultured on the prepared hydrogels.

**Conclusions:**

In conclusion, this study provides evidence that 3D CA/AA/TP composite hydrogels seeded by rMSCs in hypoxic conditions have great potential to improve wound healing.

**Graphical abstract:**

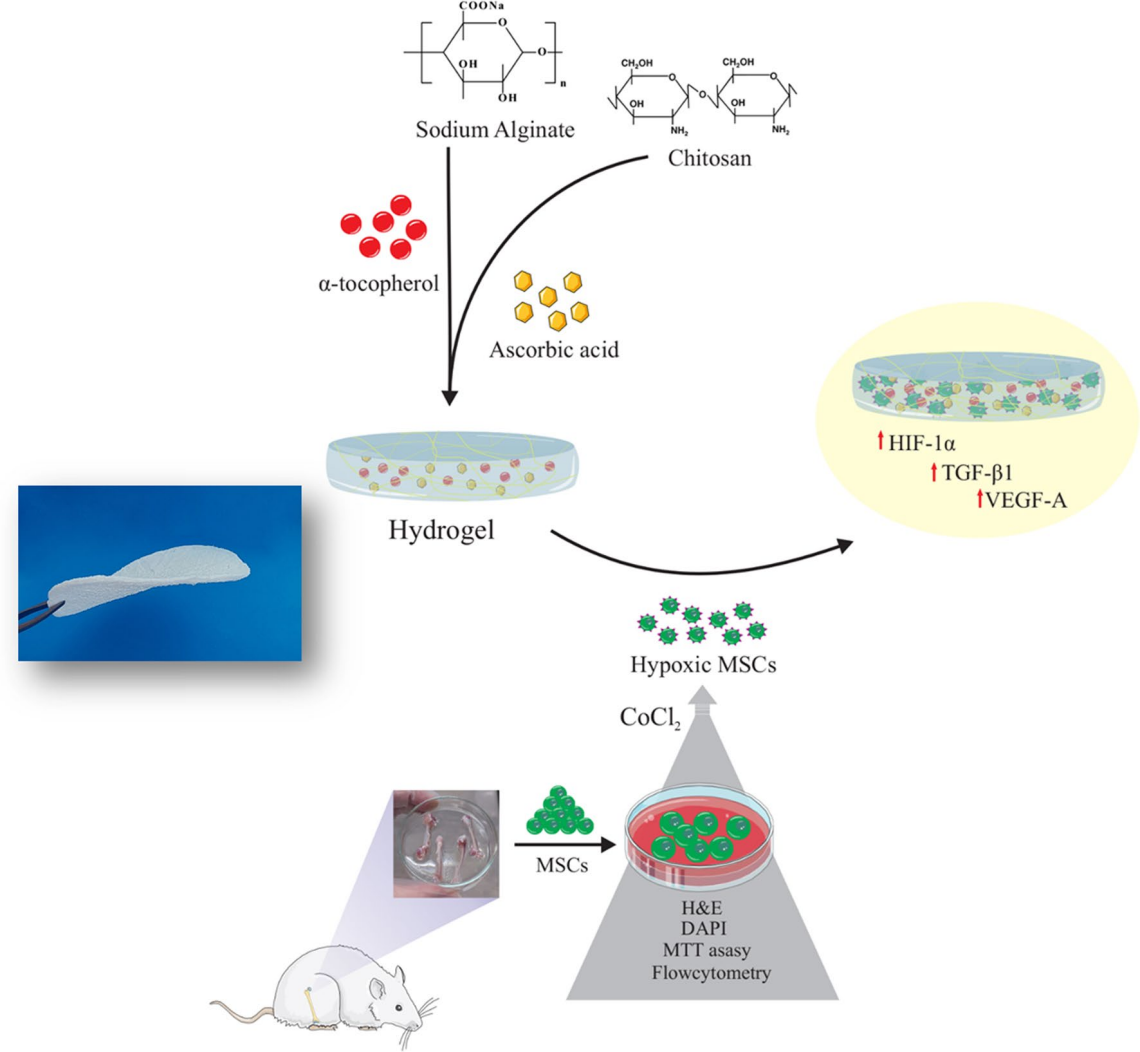

## Introduction

The skin is the largest and most vital organ in the human body. Wounds result from several factors, including surgery, trauma, burns, and diabetes [1–4]. The synchronized collaboration of cellular migration and proliferation, collagen synthesis, angiogenesis, and tissue regeneration at the wound site is necessary for proper wound healing [2]. If the normal process of wound healing is disrupted and prolonged, it leads to chronic non-healing wounds, such as those found in diabetic patients and keloid scars [3]. Despite various therapeutic intervention efforts to promote wound healing, researchers still face difficulties in identifying the most effective treatment methods. To enhance the inherent wound-healing process, several novel drugs and dressing-based therapies have been developed in recent years [2, 3, 5]. A suitable wound dressing material must have sufficient mechanical strength and flexibility and the ability to adhere neither to the wound nor the surrounding tissue [2, 6]. Among the commercialized versions of conventional wound dressings currently available in the market, cotton wool and gauze have taken up the majority of the market share [2]. However, using foams, hydro-fibers, superabsorbent materials, and alginates as advanced dressings has witnessed a remarkable increase, as these types of dressing provide a moist wound environment [2, 7–9]. Hydrogels are hydrophilic three-dimensional polymer networks that are insoluble in water and can absorb large amounts of water [10]. Nowadays, hydrogel biomaterials derived from natural polysaccharides have been widely utilized in tissue engineering and regeneration [11, 12]. These organic materials are non-toxic, biocompatible, biodegradable, and renewable [2, 13, 14]. It can be said hydrogels have the desirable characteristics of an ideal wound dressing, which makes them a suitable choice for wound-healing applications [1]. They show a diverse range of mechanical properties and have the potential to reduce pain by increasing moisture in the wound area and cooling the wound surface [1, 15]. Due to the adjustable physical properties of hydrogels, including degradability and swelling, they can regulate the release rate of drugs. Consequently, they serve as valuable drug delivery systems in clinical applications [1, 15]. Today, several wound care products are available on the market for clinical use, including Tegasorb, DuoDERM, Comfeel, Granuflex, and Aquacel. However, these products are not recommended for infected wounds. Other products that are more advanced in wound healing, such TransCyte, Integra Omnigraft, Myskin, Hyalograft, Laserskin, and EZ Derm, are not appropriate because of their potential to cause infections and immunogenicity, as well as their high cost [2]. To overcome these problems, researchers used chitosan, which has suitable properties. The presence of free amino groups in the molecular structure of chitosan has made this polymer insoluble in water. Still, upon protonation of these amino groups, it becomes soluble in water under acidic pH conditions [2]. Therefore, it can be concluded the presence of free amines and *N*-acetyl groups is essential in defining the solubility of chitosan. There are many uses of chitosan in the field of wound healing and tissue engineering applications due to its various inherent properties such as antimicrobial activity, high water absorption capacity, biodegradability, and biocompatibility, also it is fully FDA-approved [2, 16]. Insufficient flexibility and its tendency to break down into smaller pieces are the disadvantages of chitosan [1]. Alginate can serve as a polyanionic polymer with the ability to interact with the amino groups of chitosan to enhance the poor mechanical properties and drug-delivery capabilities of chitosan. Similar to chitosan, alginate has desirable properties such as swelling, biocompatibility, and biodegradability and is also FDA-approved. The efficacy of tissue regeneration is highly dependent on achieving biomechanical compatibility. Due to its hydrophilic nature, alginate absorbs wound exudate and maintains a moist microenvironment. Previous findings suggest that chitosan-alginate hydrogel plays a pivotal role in accelerating wound healing due to its inherent antimicrobial activity [1, 2, 15, 17].

Conversely, studies show that the adequacy of micronutrient nutrition is essential for promoting optimal skin health and aesthetics, and this claim is supported by examining vitamin deficiency conditions. Ascorbic acid (AA) plays a role as an essential cofactor in the metabolic pathways of carnitine and catecholamine, facilitates dietary iron absorption, and participates in collagen biosynthesis [18]. The human body does not have the ability to synthesize ascorbic acid, so this nutrition is obtained through the consumption of fruits and vegetables [18]. Ascorbic acid exhibits antioxidant properties, acts as a scavenger, prevents scar formation and skin damage, and also improves the performance of the skin barrier and moisture retention [19, 20]. This micronutrient represents a remarkable combination that supports wound healing by means of stimulating collagen synthesis and cellular proliferation [20]. Proline residues on procollagen need ascorbic acid for hydroxylation, thereby signifying its indispensability for the formation of the mature collagen triple helix. The lack of a stable triple helix structure induces detrimental to the structural integrity of the skin, blood vessels, mucous membranes, and bone tissue [18].

Another important bioactive agent in the process of wound healing is α-Tocopherol (TP), and it is the biologically active variant of vitamin E in the mammalian body [2]. This bioactive agent protects the polyunsaturated lipids and cell membrane against oxidative stress [2]. Despite the wide-ranging significance of α-Tocopherol, researchers have done many investigations to explore the antioxidant properties of α-Tocopherol. Some researchers believe that α-Tocopherol preserves the structural integrity of human body cells by regulating the cell signaling pathways and also prevents wounds from infectious agents methicillin-resistant Staphylococcus aureus (MRSA) by modulating the expression of connective tissue growth factors [2].

In addition to the above, the regulation of the wound healing process, which includes epithelialization, angiogenesis, granulation tissue formation, and wound contraction, is under the control of hypoxia-induced factor (HIF-1α). Under normal conditions, prolyl hydroxylase domain (PHDs) proteins catalyze the oxygen-dependent hydroxylation of HIF-1α, resulting in a decrease in the steady-state level of HIF-1α. Also, in the context of hyperglycemia in individuals with diabetes, the stability of HIF-1α is reduced. Consequently, the expression of the target gene of HIF-1α is inhibited, which ultimately leads to disruption of the progression of the wound healing process in these patient populations. As a result, it can be said that HIF-1α is effective in the treatment of complications associated with diabetic ulcers [21]. Investigations have demonstrated that hypoxia is not only a common pathological phenomenon in wounds but also has a pivotal function in regulating cellular bioactivity [22].

Based on the characteristics described for chitosan and alginate as natural polysaccharides, along with the acknowledged efficacy of biological agents in wound healing (ascorbic acid and α-tocopherol), it seems that hydrogels derived from these materials either individually or in combination exhibit high potential in determining the fate of stromal cells involved in wound-healing processes under hypoxic conditions. The current investigation aimed to examine the possible effects of biocompatible polysaccharide composite hydrogels enriched with AA and TP on the behavior of rMSCs and subsequent wound healing under hypoxic conditions.

## Materials and methods

### Materials

Sodium alginate was obtained from CARLO ERBA Reagent (France). Chitosan (medium molecular weight) and 3-(4,5-Dimethylthiazol-2-yl)-2,5-diphenyltetrazolium bromide (MTT) were supplied by Sigma-Aldrich Co. *α*-Tocopherol and ascorbic acid were purchased from Sigma (USA). Dulbecco’s modified Eagle's low-glucose medium (DMEM) was purchased from GIBCO (Life Technologies). Trypsin and penicillin–streptomycin (Pen/Strep) were obtained from Bio idea, and fetal bovine serum (FBS) was purchased from AnaCell. Acetic acid (glacial) 100% was purchased from Merck.

### Preparation of Chi/Alg/ AA/TP hydrogel

The chitosan/alginate (CA) hydrogels containing CA microparticles and bioactive agents (ascorbic acid and α-tocopherol) were prepared by ionic gelation according to the following steps: 1% glacial acetic acid to dissolve chitosan at 20–25 °C overnight used. Alginate was dissolved in deionized water at 20–25 °C overnight. Then, equal proportions of alginate and chitosan were blended together at room temperature for 25 min and a 1:1 chitosan-alginate solution (1% chitosan and 1% alginate) was obtained. Afterward, 400 IU of α-Tocopherol was added to a 10 ml chitosan and alginate mixture to prepare the drug-containing hydrogel. Once again, 100 μM ascorbic acid was added to the chitosan and alginate mixture. Finally, 400 IU α-Tocopherol and 100 μM ascorbic acid were added to 10 ml chitosan and alginate and vigorously stirred to form a uniform solution and microparticles. All prepared hydrogels were immersed in 1% w/v CaCl_2_ solution in deionized water for 10 min to form cross-linking. Hydrogels were then immersed in PBS (pH = 7.4) for 10 min to remove unbound cross-linkers. The samples were then lyophilized in a freeze dryer for 48 h at − 50 °C. In the present study, four groups of hydrogels, including chitosan/alginate (CA), chitosan/alginate/ascorbic acid (CA/AA), chitosan/alginate/α-tocopherol (CA/TP), and chitosan/alginate/ascorbic acid/α-tocopherol (CA/AA/TP), were prepared.

### Hydrogel characterization

#### Morphological properties

To investigate the surface morphology and porosity of composite hydrogels and the CA microparticles incorporated in them, the freeze-dried samples, including CA, CA/AA, CA/TP, and CA/AA/TP, were analyzed by scanning electron microscope (MIRA3 FEG-SEM, Tescan, Brno, Czech Republic) at an accelerating voltage of 30 kV and resolution of 1 nm. To this end, the samples were cut into 1cm × 1cm dimensions, coated with gold, and then evaluated with an SEM microscope.

#### Fourier transform infrared spectroscopic analysis (FTIR)

Infrared spectroscopy was used for the chemical characterization of composite hydrogels, the molecular composition of AA and TP, and chemical bonds. The samples were prepared as compressed KBr disks at room temperature and placed in a magnetic holder. Finally, FTIR analysis of freeze-dried samples, including pure alginate and chitosan, CA, CA/AA/TP, as well as a powder form of TP and AA, was done using a Thermo Scientific Nicolet iS5 FTIR spectrometer in the range between 4000 and 400 cm^−1^.

#### Wettability measurement

The surface hydrophilicity of composite hydrogels was investigated by measuring the contact angle. A contact Angle Meter (IFT-CA, CA-ES20, Iran) was used to investigate freeze-dried hydrogel’s wettability. In this way, the samples were cut into dimensions of approximately 2 × 3 cm^2^, distilled water was dripped on the surface, and the contact angle of the water formed at the interface between the water and the sample surface was recorded.

#### Weight loss analysis

To determine in vitro degradation rate, freeze-dried hydrogels (including CA, CA/AA, CA/TP, CA/AA/TP) were cut and weighed. The solid samples were immersed in phosphate-buffered saline (PBS) with pH 7.4 and placed in a 37 °C incubator. Then, at intervals of 1, 2, 3, 7, 14, and 21 days, they were removed from the buffer and dried at 37°C, and then their dry weight was measured. To measure the degree of degradation, the obtained weights were placed in the following formula (1) [2]:

1 $${\text{Weight}}\,\,{\text{loss }}\,\left( \% \right)\, = \,\frac{W0 - W1}{{W0}} \times 100$$

where *W*_0_ represents the initial dry weight of the hydrogel composite and *W*_1_ is the final dry weight.

#### Swelling behavior

To evaluate the fluid uptake of hydrogels, including CA, CA/AA, CA/TP, and CA/AA/TP samples, freeze-dried samples were cut, their dry weight was recorded, and then they were immersed in PBS buffer (pH = 7.4) and were placed in a 37 °C incubator. After predetermined times (2, 4, 24, 48, and 72 h), the samples were removed from the buffer, and after removing the excess water from the swollen hydrogels using filter paper, they were quickly weighed. The swelling capacity percentage was obtained using Eq. (2) [2]:

2 $${\text{Swelling}}\, {\text{ratio}}\, \left( \% \right)\, = \,\frac{W2 - W1}{{W1}} \times 100$$

where *W*_2_ represents the swollen weight of the composite hydrogels and *W*_1_ is the dry weight.

#### Release study

UV–visible spectroscopy was used to investigate the release of α-TP and AA from composite hydrogels in vitro. CA hydrogel (15 mg) containing 9 mg TP, as well as CA hydrogel containing 100 μM AA, was placed separately in a falcon tube containing 20 ml PBS buffer (pH 7.4) and incubated in a shaker incubator (50 rpm) under temperature 37 °C (optimal concentration of drugs was determined based on articles [2, 23]). At specified time intervals (6, 12, 24, 48, 72, and 120 h), the buffer containing the released drug was changed with 20 ml of fresh buffer. The absorbance intensity of the buffer containing the released drug for TP and AA was recorded at *λ*_max_ = 290 nm and *λ*_max_ = 265 nm, respectively, by UV–Vis spectrophotometer. Equation 3 was used to determine the release rate of TP and AA [2].

3 $${\text{Drug}} \,\,{\text{release}} \,\left( \% \right)\, = \,\frac{{{\text{amount}}\,\, {\text{of}}\,\,{\text{released}}\,\, {\text{drug}}\,\,{\text{in}}\,\,{\text{buffer}}}}{{{\text{amount}}\,\,{\text{of}}\,\,{\text{loaded}}\,\,{\text{drug}}\,\,{\text{into}}\,\,{\text{the}}\,\,{\text{hydrogel}} }} \times 100$$

#### Hemolysis study

To investigate the blood compatibility of freeze-dried hydrogels, including CA, CA/AA, CA/TP, and CA/AA/TP, first, all samples were cut into 3 mg pieces and placed in 2-ml microtubes. Then, 200 µl of fresh rat blood were collected in citrated tubes, and 800 µl of normal saline solution was added to the samples. For the positive control group, 800 µl of deionized water was added to 200 µl of blood, and for the negative control group, 800 µl of normal saline was added to 200 µl of blood. After incubation at 37 °C for 1 h, the samples were centrifuged for 10 min at 3000 rpm, and then the absorbance of the supernatant was obtained at *λ*_max_ = 540 nm using a UV–vis spectrophotometer. The percentage of hemolysis was obtained with Eq. (4) [24]:

4 $${\text{Hemolysis}}\,\left( \% \right)\, = \,\frac{{OD_{{{\text{Sample}}}} - OD_{{{\text{Negative}}\,\,{\text{ control}}}} }}{{OD_{{{\text{Positive}}\,\,{\text{control }}}} - OD_{{{\text{Negative}}\,\,{\text{control}}}} }} \times 100$$

### Cell culture experiments

#### Isolation of rMSCs

Rat bone marrow mesenchymal stem cells (rMSCs) were isolated from the femur and tibia of male Wistar rats (12 weeks old). The animals were maintained in accordance with the guideline set forth by the local Animal Care Committee (IR.TBZMED.VCR.REC.1400.034) in the Animal House of Tabriz University of Medical Sciences. To isolate bone marrow mesenchymal stem cells (BM-SCs), the rats were first euthanized with high doses of ketamine and xylazine, femur and tibia bones were harvested, and the epiphysis was aseptically cut off. Then, bone marrow was flushed into the cell culture flasks by Low-Glucose Dulbecco’s Modified Eagle’s Medium (DMEM-LG; Gibco, Germany), supplemented with 10% (v/v) fetal bovine serum (FBS; Anacell, Iran) and 1% pen-strep (Biosera, Sussex, UK). The flasks were placed in an incubator at 37 °C in a humidified atmosphere of 95% air and 5% CO_2_. After 24 h, the supernatant was aspirated, and adherent cells were washed twice with pH 7.4 PBS. Finally, cells were grown in a standard growth medium until reaching 80% confluence.

#### Characterization of rMSCs by flowcytometry

To characterize rMSCs, in the third passage, the cells were trypsinized, and after washing with PBS, ten tubes were selected, and the cells were separately incubated with anti-rat monoclonal antibodies containing anti-CD34, -CD45, -CD73, and -CD105 for 30 min at 4 °C. Then, for washing, 500 µl of the PBS solution was added to the tubes and centrifuged for 5 min at a speed of 1500 rpm. After removing the supernatant, 250 µl of PBS was added to the cells, and analysis was performed using BD FACS Calibur (BD biosciences, San Jose, CA, USA).

#### Cell viability studies

To evaluate biocompatibility, hydrogels were first cut and sterilized with 70% v/v alcohol. Then, they were washed three times with PBS and transferred to 96-well plates. Next, rMSCs were cultured on the hydrogels at a density of 1 × 10^4^ cells in DMEM supplemented with 10% (v/v) FBS, 1% of pen-strep and incubated in a humidified incubator (37 °C, 5% CO2). After 72 h, the supernatant was removed from the 96-well plates, and 0.2 ml MTT (0.5 mg/ml) was added to each well and incubated for four hours in an incubator at 37 °C. MTT was then replaced with 0.1 ml DMSO for 30 min. Finally, absorbance was measured by an ELISA reader at 570 nm to determine cell viability.

#### H&E staining

Hematoxylin and eosin (H&E) staining was used to show the presence of cells inside the hydrogels. In this regard, rMSCs were cultured on the CA hydrogels. After 3, 5, and 7 days, cell-containing hydrogels were fixed by 10% formalin solution, and after washing with PBS, the samples were immersed in Xylene, 100% ethanol, 95% ethanol, 80% ethanol, and deionized H_2_O, respectively. Finally, the samples were stained with hematoxylin and eosin and examined using light microscope images.

#### DAPI staining

DAPI staining was used to stain the nuclei of living cells in hydrogels. In brief, CA hydrogels were placed in 6-well plates and sterilized with 70% v/v alcohol. After washing with PBS, rMSCs were cultured onto the hydrogels, and after 24 and 72 h, the culture medium was removed. Then, cell-containing hydrogels were washed with PBS and incubated with 3.7% formaldehyde at room temperature for 10 min. Next, the cells were washed 1–3 times with PBS. After removing the buffer, 2 mL DAPI staining solution was added to each well and incubated at room temperature in the dark to bind to AT-rich DNA. After 15 min, the stain solution was removed, and the stained samples were washed with PBS. Finally, cell-containing hydrogels were imaged by an Olympus BX50 fluorescence microscope.

#### Hypoxia induction and real-time PCR

MSCs were cultured onto the hydrogels in separate groups for three days. Then, to apply hypoxia, the cells were exposed to a medium containing 100 µl of CoCl_2_ for 8 h. Then, the supernatant of all groups was removed, and Trizol reagent (MXcell-Tehran-Iran) was used for RNA extraction. For the cDNA synthesis, 1ng of RNA was removed from each group and subjected to cDNA synthesis according to the cDNA synthesis kit protocol (Yekta Tajhiz Azma Iran). Real-time PCR was used for looking at gene expression of hypoxia-inducible factor 1α (HIF-1α), vascular endothelial growth factor-A (VEGF-A), and transforming growth factor- β1 (TGF-β1) using the gene-specific primers in Table 1. β-actin was considered a housekeeping gene. Samples include N-CA in normoxic condition (N) as a control group and H-CA, H-CA/AA, H-CA/TP, and H-CA/AA/Tp in hypoxic condition (H) to monitor the expression of VEGF-A and TGF-β1. Also, N-CA and H-CA were used to monitor the expression of HIF-1α. Method 2(-Delta Delta C (T)) was used to analyze relative changes in gene expression in real-time PCR experiments [25].

**Table 1:** Primer sequences used for real-time quantitative PCR (q-PCR)

Gene	Primer sequence (5′–3′)	Product length (bp)
HIF-1α	Forward GTGCCCCTACTATGTCGCTT	137
	Reverse CTCATCCATTGACTGCCCCA	
VEGF-A	Forward GGCTTTACTGCTGTACCTCCA	147
	Reverse CACACAGGACGGCTTGAAGA	
TGF-β1	Forward TCCATGACATGAACCGACCC	142
	Reverse TGCCGTACACAGCAGTTCTT	
β-actin	Forward TGACAGGATGCAGAAGGAGA	104
	Reverse TAGAGCCACCAATCCACACA	

### Statistical analysis

The mean ± standard deviation of each sample group represents the results. GraphPad Prism software (version 8.3.0, USA) was used to perform statistical analysis based on a two-way ANOVA and Tukey post hoc analysis for all tests except the real-time PCR assay. T-test and one-way ANOVA analysis were used for real-time PCR assay. The experiments were performed in triplicate.

## Results

### Morphological and ultrastructural characterization of hydrogels

**Fig. 1:**
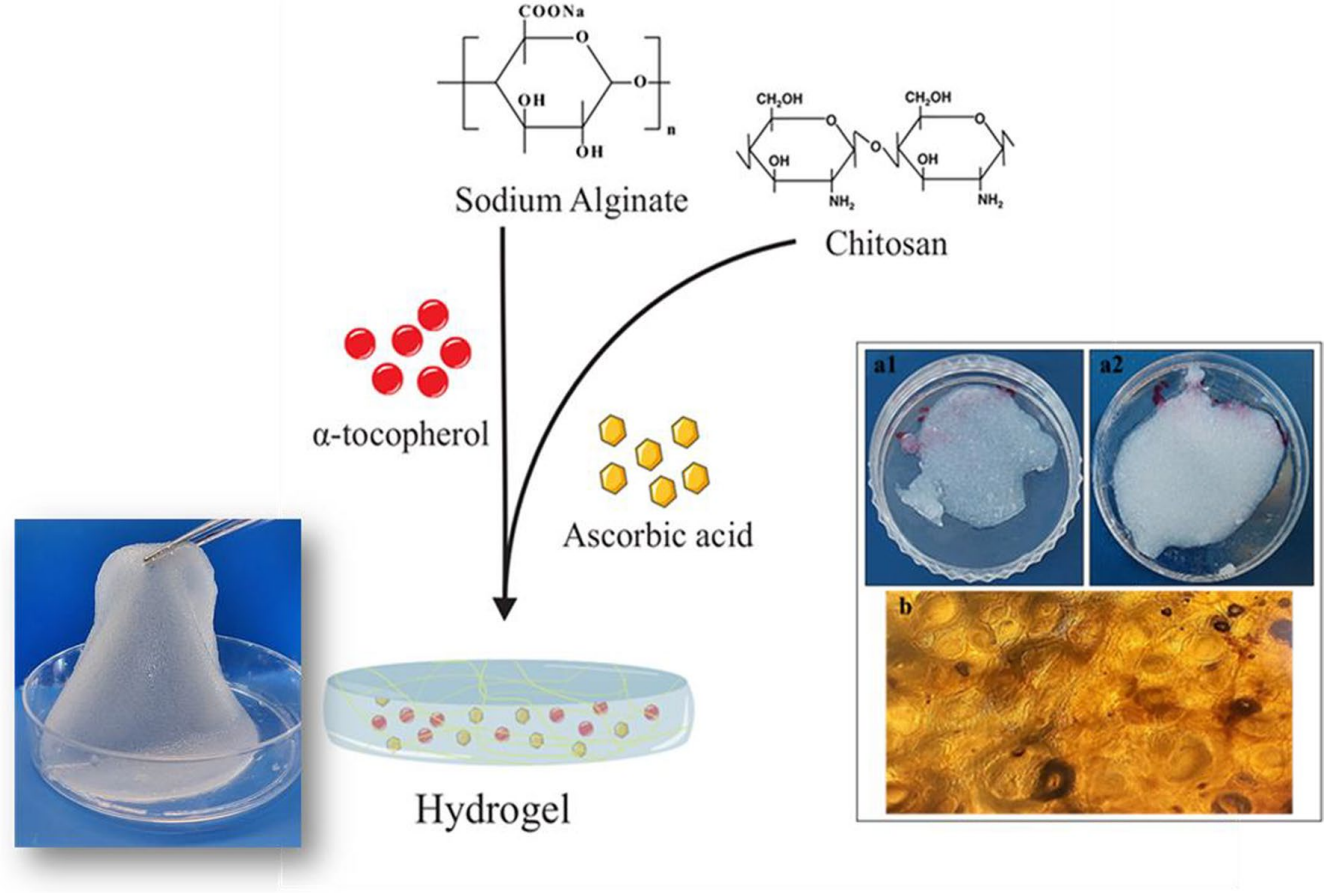
Appearance of the hydrogels: **a1** AA-loaded hydrogel (left), **a2** TP-loaded hydrogel (right). **b** The inverted light microscopy image of the hydrogel after cross-linking with CaCl2

Figure 1a shows the appearance of blank hydrogel and drug-loaded hydrogel. The blank hydrogel and hydrogel containing AA were transparent, but after adding TP, the hydrogel turned white and opaque. It also showed that the TP was homogeneously distributed into the hydrogel. Figure 1b shows the inverted light microscopy image of the hydrogels after cross-linking with CaCl_2_. In the image, the networks created by cross-linker can be seen.

**Fig. 2:**
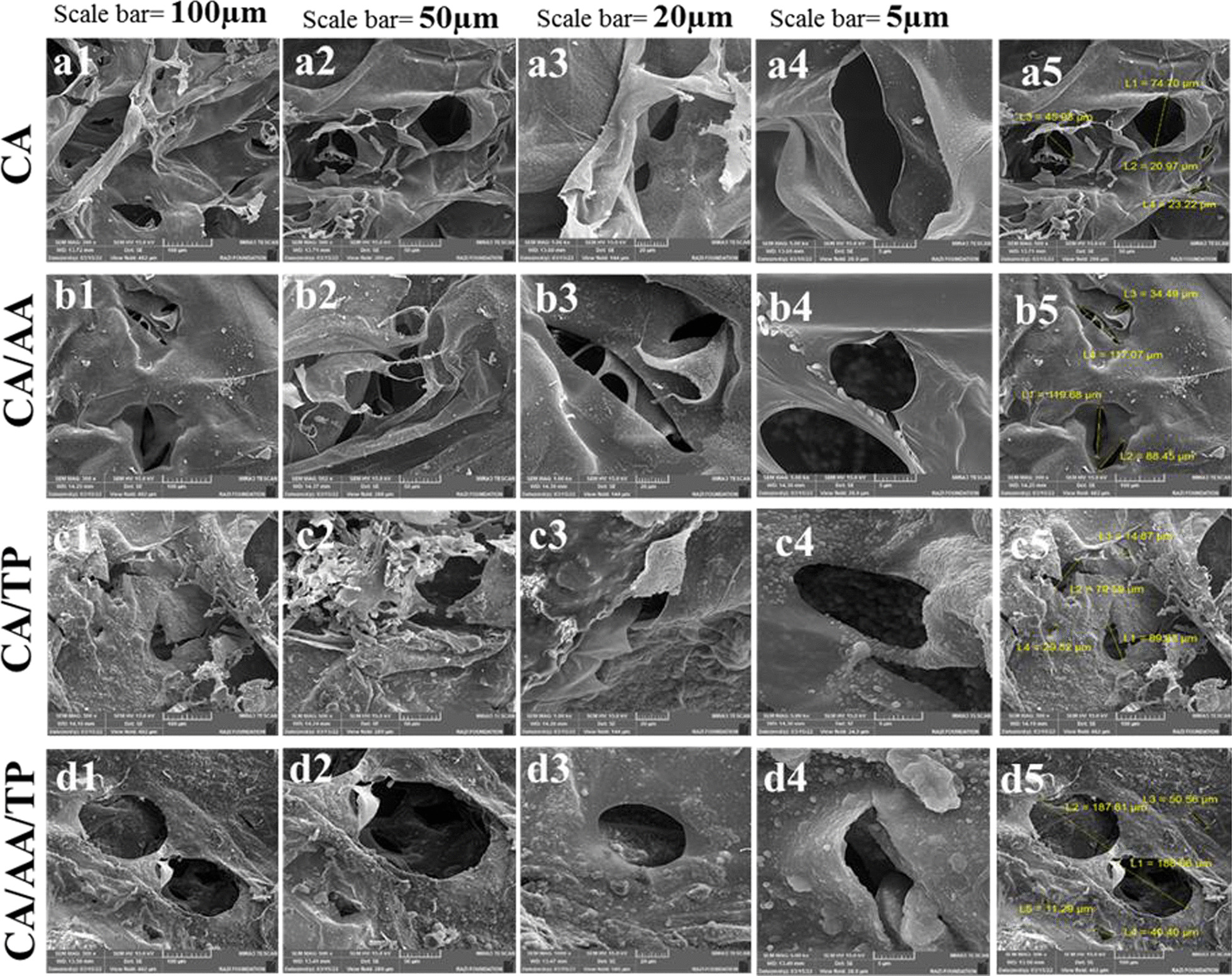
SEM images of hydrogels: **a1**–**a5** CA hydrogel, **b1**–**b5** CA/AA hydrogel, **c1**–**c5** CA/TP hydrogel, **d1**–**d5** CA/AA/TP hydrogel, with magnification 100, 50, 20, and 5 µm. **a5**, **b5**, **c5**, and **d5** show the pore sizes of CA, CA/AA, CA/TP, and CA/AA/TP hydrogels, respectively

SEM images of fabricated composite hydrogels (CA, CA/AA, CA/TP, and CA/AA/TP) with magnifications of 100, 50, 20, and 5 µm and their pore size are shown in Fig. 2. Based on the SEM images, the freeze-dried hydrogels were highly porous with irregular interconnected pores, and the average pore dimensions were calculated by Image-J Software Version 1.8.0. The average pore dimensions for CA, CA/AA, CA/TP, and CA/AA/TP hydrogels were 41.20, 89.92, 53.47, and 95.7, respectively. SEM images (Fig. 2) showed that AA-loaded hydrogels have the largest average pore size because membrane formation is negatively affected by the addition of AA to chitosan, and the degradation of the pore wall increases the pore size [26]. However, pore size was not significantly different between the TP-loaded hydrogel and the blank CA samples. All samples studied in this research have pore sizes ranging from 20 to 188 μm.

**Fig. 3:**
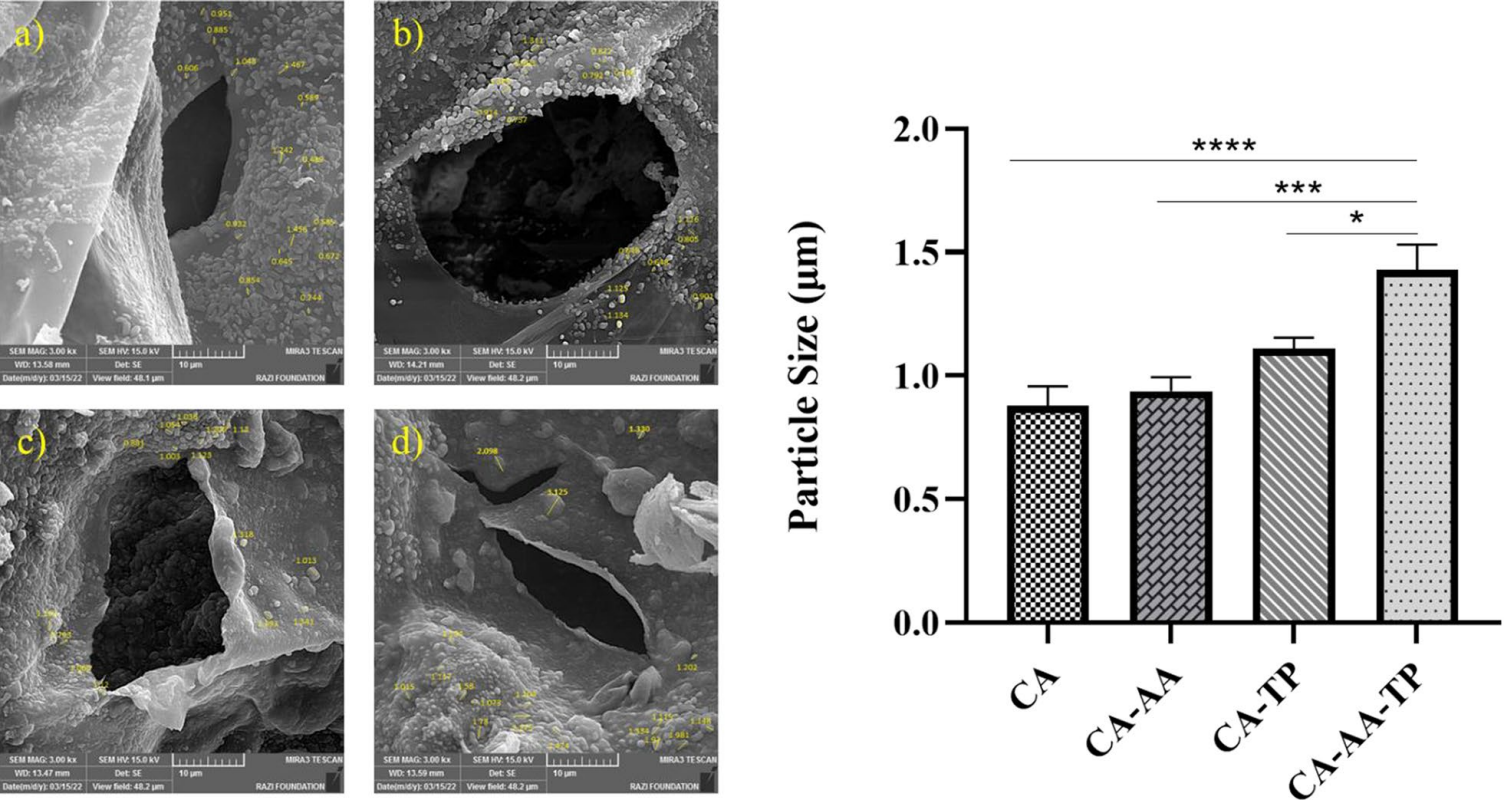
SEM images of CA microparticles: **a** CA hydrogel, **b** CA/AA hydrogel, **c** CA/TP hydrogel, **d** CA/AA/TP hydrogel, at 10 μm magnification. Histogram shows the size of CA particles in CA, CA/AA, CA/TP, and CA/AA/TP hydrogels. One-Way ANOVA and Tukey post hoc analysis, ****p < .0001, ***p < .001, and *p < .05

On the other hand, in Fig. 3. SEM images of CA microparticles in composite hydrogels can be seen. Also, the size of CA particles in CA, CA/AA, CA/TP, and CA/AA/TP composite hydrogels was calculated by Image-J software. Analysis of the size of CA microparticles showed that AA and TP loaded in composite hydrogels strongly interacted with polymers and subsequently had a proper distribution in CA particles. The maximum particle diameter was observed in the composite hydrogel loaded with both AA and TP (*p* < 0.0001).

### FTIR spectroscopy

**Fig. 4:**
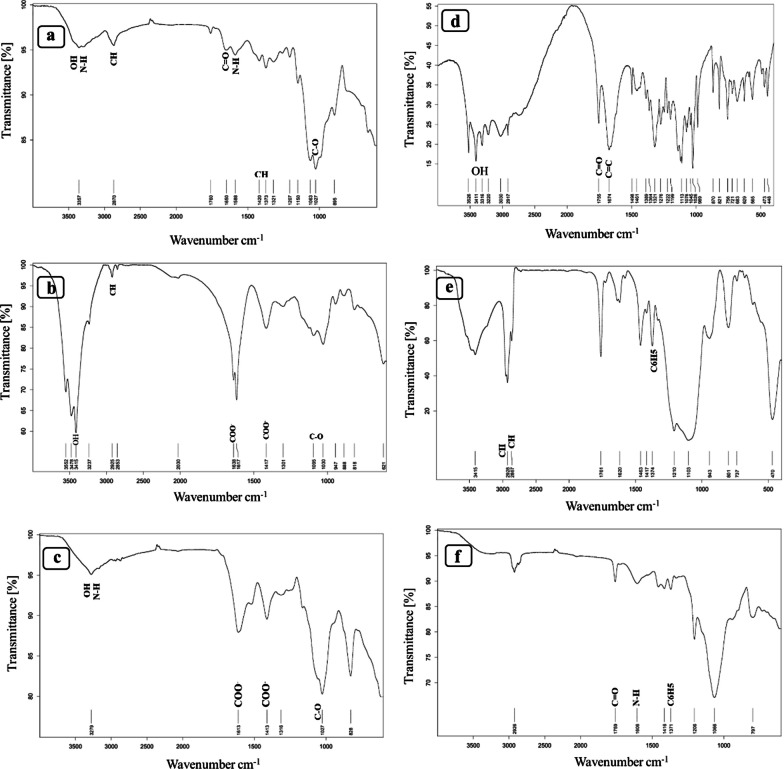
FTIR spectrum of chitosan and alginate pure and their combination as CA (**a**–**c**) and FTIR spectrum of AA, TP pure and their combination with CA (**d**–**f,** respectively)

The FTIR spectrum is shown in Fig. 4. The spectra are related to functional groups in pure alginate, chitosan, and hydrogels composed of these two, as well as pure AA, TP, and hydrogels loaded with AA and TP. Figure 4a shows the spectrum of chitosan. Absorption at 3357 cm^−1^ is related to the bending and stretching vibration of overlapping amine and hydroxyl groups (-NH and –OH). The peak at 2870 cm^−1^ represents the stretching vibrations of the polymer backbone's asymmetric and symmetric C–H groups. Other peaks at 1650 cm^−1^ correspond to the carbonyl (C=O stretch of amide I) group, and a band at 1588 cm^−1^ is related to the –NH group representing the amine groups in chitosan. The low-intensity peak observed at 1420 cm^−1^ and 1373 cm^−1^ are indicating the CH_2_ groups. The band at 1027 cm^−1^ is linked to the stretching vibrations of COC groups [27–30]. Figure 4b corresponds to the spectrum of sodium alginate. The peak at 3415 cm^−1^ is related to the vibration of a hydroxyl group (–OH). Observed bands in 2925 cm^−1^ refer to stretching vibrations of alkyl groups (C–H,) and other peaks at 1638 cm^−1^ and 1417 cm^−1^ are linked with the asymmetric and symmetric stretching vibrations of carboxyl groups (–COO), respectively, and the peak at 1095 cm^−1^ and 1030 cm^−1^ is attributed to C–O–C group [31–33]. Figure 4c shows the FTIR spectrum of the correlation of chitosan and alginate in the hydrogel composition. The carboxyl groups (–COO) originating from sodium alginate, which peaked at 1638 cm^−1^ and 1417 cm^−1^, were shifted to the bands of 1613 cm^−1^ and 1413 cm^−1^ with lower intensity and in band 1613 cm^−1^ overlapped with carbonyl (C=O) group of chitosan. In addition, amine and hydroxyl (–NH and –OH) groups of chitosan (3357 cm^−1^) and hydroxyl (–OH) group of alginate (3415 cm^−1^) have both moved to 3279 cm^−1^. Figure 4d corresponds to the FTIR spectrum of pure AA. The peaks in the range of 3526–3220 cm^−1^ are related to hydroxyl groups (-OH) in the AA structure. The peak at 1755 cm^−1^ and 1674 cm^−1^ represents the stretching vibrations of C=O and C=C bands, respectively [34]. Figure 4e is related to the structure of pure TP, where the symmetric and asymmetric vibration of –CH_3_ and –CH_2_ groups and the phenyl group can be seen at peaks 2928 cm^−1^, 2867 cm^−1^, and 1374 cm^−1^, respectively [35]. The spectrum resulting from the combination of AA in the CA is shown in Fig. 4f, which shows that after the combination of AA and TP with CA, the peak intensity of the 3500–2600 cm^−1^ regions has decreased. The peak of AA has been shifted from 1755 to 1759 cm^−1^, and its intensity has also decreased significantly. Also, the peak at 1674 cm^−1^ has disappeared. It can be assumed that the band appearing in 1597 cm^−1^ is due to the protonation of -NH_2_ groups on chitosan chains by AA. The peak at 1371 cm^−1^ is related to the phenyl group of TP, which has been shifted from 1374 cm^−1^ to this region. Also, due to the overlapping of CA, AA, and TP peaks in the region of 1400–1600 cm^−1^, the intensity of the peak in this region has been decreased [36, 37].

### Wettability measurement

**Fig. 5:**
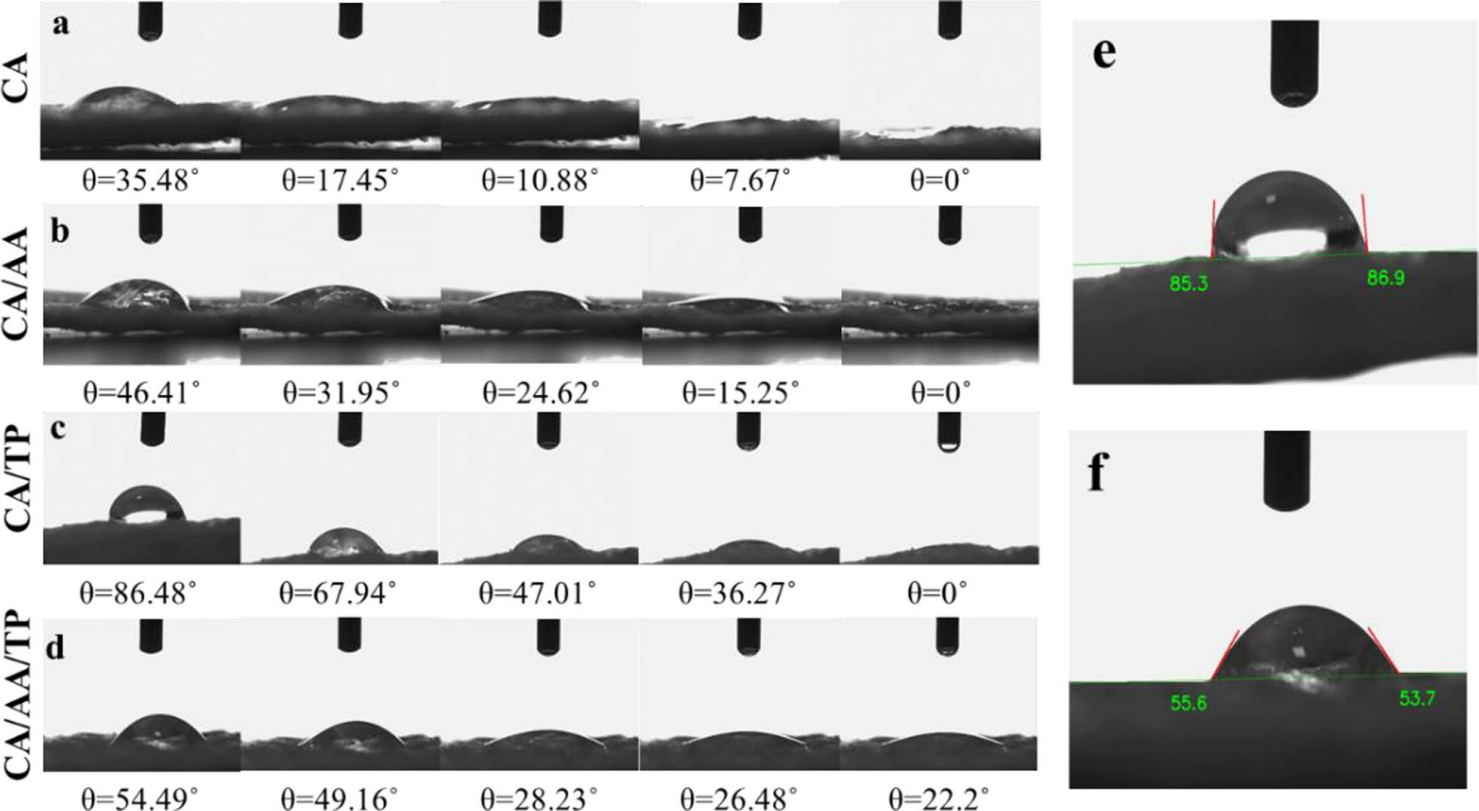
The pictures of the dynamic contact angle on **a** CA, **b** CA/AA, **c** CA/TP, and **d** CA/AA/TP composite hydrogels at different time points. e and f show the calculated contact angles for samples CA/TP and CA/AA/TP, respectively

Hydrophobicity or hydrophilicity is often characterized by surface morphology and free energy. The contact angle of the surface with water can be used to check it [38]. The wettest surfaces have low values (less than 20°), and hydrophobic surfaces have high contact angle values (more than 90°) [38, 39]. The comparison of samples showed that CA and CA/AA samples have the lowest contact angle (almost zero degrees) (Fig. 5a–d). As a result, these two hydrogels were the most hydrophilic. When combining chitosan and alginate with TP, the water contact angle increased (*θ* = 85.3° and 86.9°) (Fig. 5e). But in the sample containing both AA and TP, the contact angle decreased (*θ* = 55.6° and 53.7°) (Fig. 5f). Since these contact angles are less than 90°, it indicates their hydrophilicity. In general, the hydrophobicity of CA/TP and CA/AA/TP composite hydrogels has increased compared to CA and CA/AA samples. The increase in contact angle may also be explained by a change in morphology that disrupts water diffusion [38]. Also, in the structure of chitosan, the intermolecular H bonds between –OH and –NH_2_ groups are broken in the presence of AA, and it can be concluded that this factor increases the hydrophilicity of CA/AA/TP hydrogels compared to CA/TP hydrogels [40].

#### Weight loss analysis

**Fig. 6:**
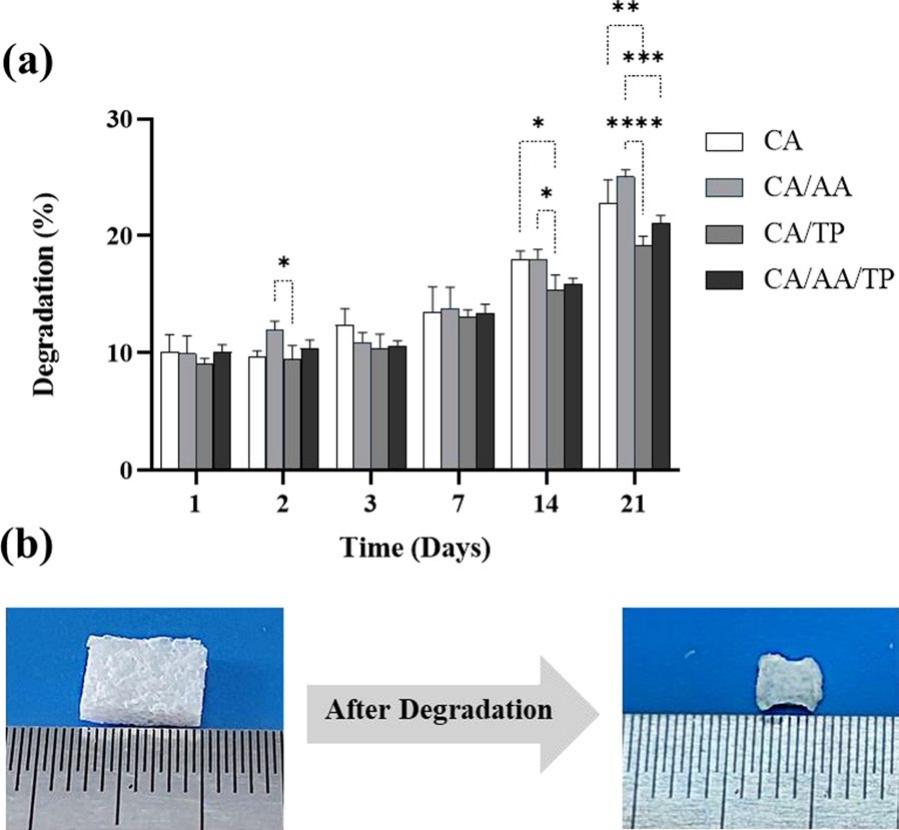
Weight loss assay over 21 days (*n* = 3). **a** Data showed that biodegradability increased when AA was added to the CA structure. Tow-way ANOVA and Tukey post hoc analysis, ****p < .0001, ***p < .001, **p < .01 and *p < .05. **b** Images of lyophilized composite hydrogels before and after degradation (*n* = 3)

Weight loss analysis was performed after incubation of the hydrogels in PBS (pH = 7.4, 37 °C) on days 1, 2, 3, 7, 14, and 21 (Fig. 6, Table 2). There was no significant difference in the mass loss of CA, CA/AA, CA/TP, and CA/AA/TP hydrogels on days 1, 2, 3, and 7 and days 14 and 21. During 21 days, the lowest degradation rate was observed in CA/TP hydrogel (19.21 ± 0.61%) due to the hydrophobic property of TP. The weight loss for CA/AA/TP and CA hydrogels was 21.09 ± 0.52% and 22.75 ± 1.65%, respectively. The CA/AA hydrogel demonstrated the highest rate of weight loss (25.02 ± 0.49%) compared to other groups.

**Table 2:** The mass loss percent of CA, CA/AA, CA/TP, and CA/AA/TP composite hydrogels during 1, 2, 3, 7, 14, and 21 days

Time (day)	1	2	3	7	14	21
CA	10.05 ± 1.23, *n* = 3	9.69 ± 0.4, *n* = 3	12.36 ± 1.17, *n* = 3	13.51 ± 1.73, *n* = 3	17.93 ± 0.64, *n* = 3	22.75 ± 1.65, *n* = 3
CA/AA	9.94 ± 1.23, *n* = 3	11.93 ± 0.63, *n* = 3	10.85 ± 0.71, *n* = 3	13.8 ± 1.47, *n* = 3	18.02 ± 0.66, *n* = 3	25.02 ± 0.49, *n* = 3
CA/TP	9.00 ± 0.4, *n* = 3	9.4 ± 1.00, *n* = 3	10.37 ± 1.02, *n* = 3	13.12 ± 0.46, *n* = 3	15.37 ± 1.05, *n* = 3	19.21 ± 0.61, *n* = 3
CA/AA/TP	10.05 ± 0.52, *n* = 3	10.37 ± 0.6, *n* = 3	10.59 ± 0.37, *n* = 3	13.34 ± 0.66, *n* = 3	15.91 ± 0.38, *n* = 3	21.09 ± 0.52, *n* = 3

### Swelling behavior

**Fig. 7:**
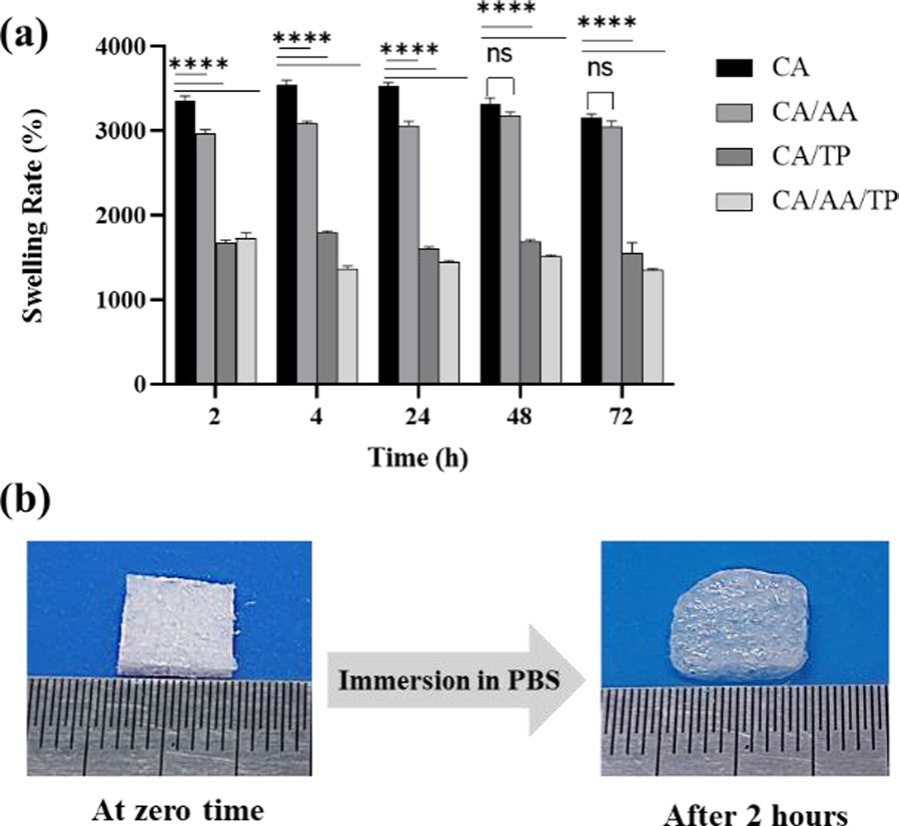
**a** Swelling rate of lyophilized composite hydrogels. Tow-Way ANOVA and Tukey post hoc analysis, ****p < .0001, and “ns” represent no significant difference between groups. **b** Images of lyophilized hydrogels before and after immersion in the PBS (*n* = 3)

One of the most critical indicators of biomaterials used in tissue engineering is their swelling ratio. Generally, the swelling of scaffolds is related to porosity, pore size, and hydrophilicity. Swollen and porous scaffolds are effective in wound healing due to better absorption of wound secretions [41, 42]. In addition, fluid flow in porous and swollen scaffolds causes the mass transfer of nutrients to cells and the elimination of metabolic waste from cells [42]. In this study, cross-linked hydrogels were obtained by 1% CaCl_2_, and the swelling rate of the lyophilized samples was measured. Table 3 and Fig. 7 illustrate the swelling rate of freeze-dried hydrogels at various periods. As shown in Fig. 7a, in the first 2 h, all samples reached their maximum swelling rate and were in a saturated state (3356.7 ± 71.69%, 2967.35 ± 61.61%, 1668.74 ± 56.66%, and 1721.98 ± 96.66% for CA, CA/AA, CA/TP, and CA/AA/TP hydrogels, respectively). These ratios remained almost constant over time. CA and CA/AA samples had more swelling than CA/TP and CA/AA/TP samples due to their high hydrophilicity [38, 39].

**Table 3:** Swelling rate of lyophilized CA, CA/AA, CA/TP, and CA/AA/TP composite hydrogels during 2, 4, 24, 48, and 72 h

TIME (H)	2 h	4 h	24 h	48 h	72 h
CA	3356.7 ± 71.69, *n* = 3	3536.9 ± 80.08, *n* = 3	3530.24 ± 53.0, *n* = 3	3310.75 ± 111.95, *n* = 3	3153.98 ± 58.51, *n* = 3
CA/AA	2967.35 ± 61.61, *n* = 3	3081.12 ± 43.26, *n* = 3	3055.78 ± 80.8, *n* = 3	3178.8 ± 62.86, *n* = 3	3049.97 ± 92.97, *n* = 3
CA/TP	1668.74 ± 56.66, *n* = 3	1784.31 ± 43.89, *n* = 3	1608.73 ± 22.4, *n* = 3	1677.3 ± 47.41, *n* = 3	1554.69 ± 170.04, *n* = 3
CA/AA/TP	1721.98 ± 96.66, *n* = 3	1360.75 ± 52.47, *n* = 3	1441.93 ± 30.0, *n* = 3	1506.24 ± 32.21, *n* = 3	1349.43 ± 29.00, *n* = 3

### Release study

**Fig. 8:**
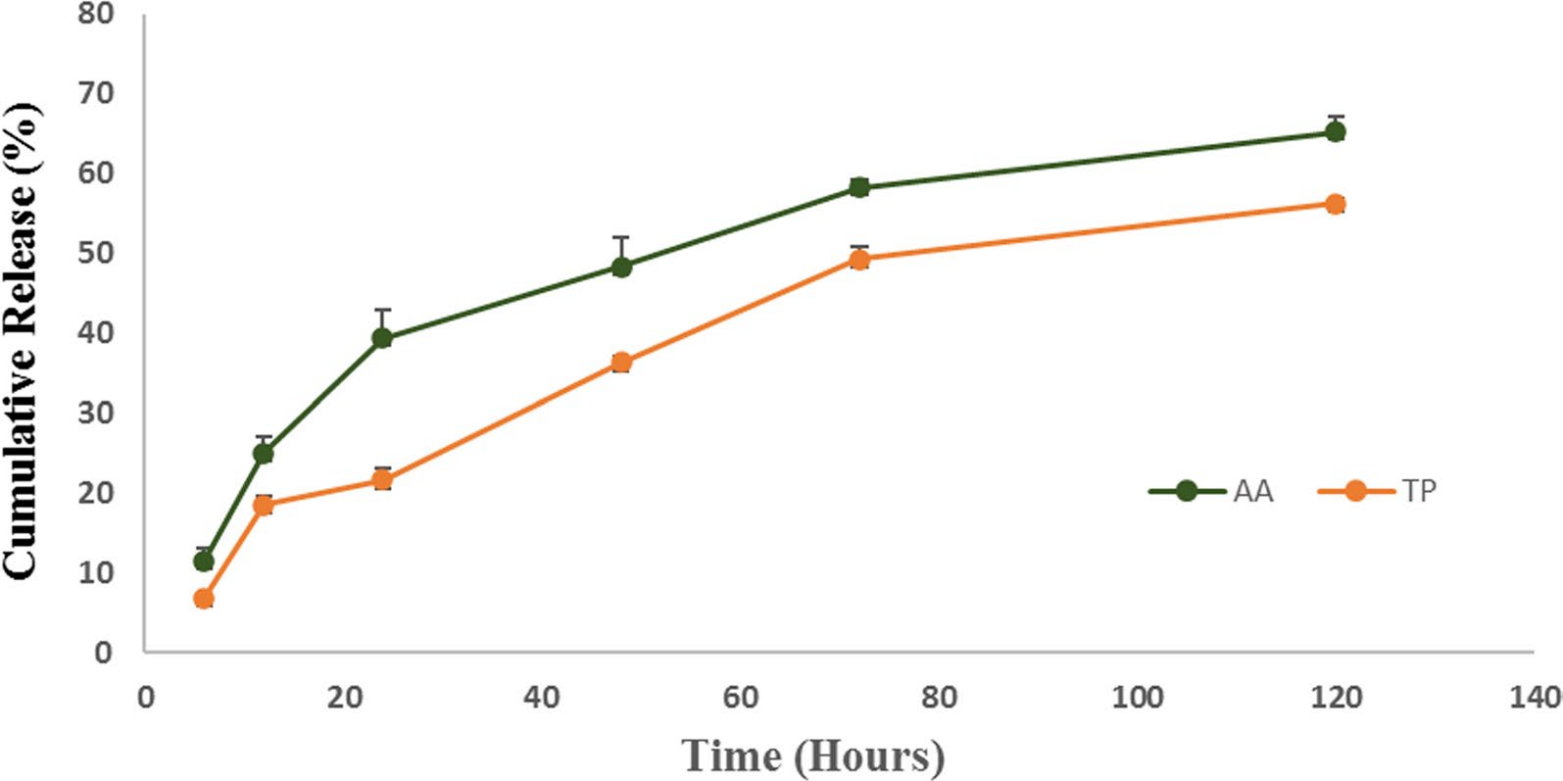
Cumulative release profiles of AA and TP loaded into CA hydrogel over a 120-h interval (*n* = 3)

Table 4 shows the release percentage of AA and TP loaded into CA during 6, 12, 24, 48, 72, and 120 h of incubation in PBS. Figure 8 also shows the sustained release of drugs. After 120 h, 65.2 ± 0.47% of AA and 56.28 ± 1.92% of TP were released. Since AA and TP were blended within the hydrogel and were not coated on the hydrogel's surface, it can be argued that the drug release is connected to the weight loss of the samples*.*

**Table 4:** Release percentage of AA and TP loaded into CA during 6, 12, 24, 48, 72, and 120 h

Time (hour)	6 h	12 h	24 h	48 h	72 h	120 h
AA release (%)	11.52 ± 0.33, *n* = 3	25.1 ± 1.14, *n* = 3	39.41 ± 1.38, *n* = 3	48.35 ± 0.66, *n* = 3	58.25 ± 1.58, *n* = 3	65.2 ± 0.47, *n* = 3
TP release (%)	6.91 ± 1.66, *n* = 3	18.5 ± 1.95, *n* = 3	21.65 ± 3.48, *n* = 3	36.32 ± 3.59, *n* = 3	49.3 ± 0.99, *n* = 3	56.28 ± 1.92, *n* = 3

### Hemolysis

**Fig. 9:**
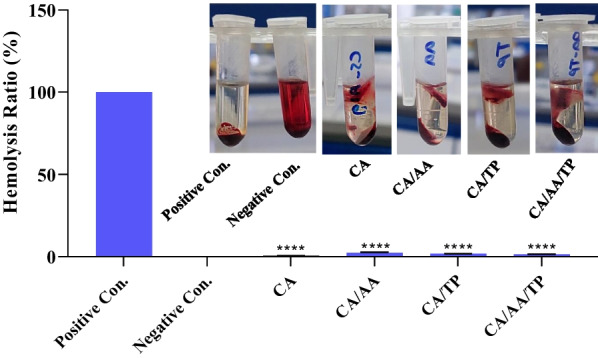
Hemolysis ratios of CA, CA/AA, CA/TP, CA/AA/TP hydrogels, positive and negative control (*n* = 3). The hemolysis ratios were CA = 0.52 ± 0.07, CA/AA = 2.46 ± 0.35, CA/TP = 1.76 ± 0.15, and CA/AA/TP = 1.36 ± 0.19. ****p < .0001

The hemolysis test is the best way to determine the biocompatibility of CA, CA/AA, CA/TP, and CA/AA/TP composite hydrogels with bloodstream RBCs. International standards define materials as blood-biocompatible if their hemolysis ratio is less than 5% [43]. The hydrogels' hemolysis ratios are displayed in Fig. 9 and Table 5. The ratio of hemolysis in all samples was less than 5% and in the best case (CA) it was reduced to 0.52 ± 0.07%, which the same or even better than other researches. Based on the findings of this study, it can be said that the hemo-biocompatibility of CA, CA/AA, CA/TP, and CA/AA/TP composite hydrogels was satisfactory.

**Table 5:** Hemolysis ratio of CA, CA/AA, CA/TP, and CA/AA/TP composite hydrogels

Samples	CA	CA/AA	CA/TP	CA/AA/TP
Hemolysis ratio (%)	0.52 ± 0.07, *n* = 3	2.46 ± 0.35, *n* = 3	1.76 ± 0.15, *n* = 3	1.36 ± 0.19, *n* = 3

### Cell culture experiments and histological analysis

**Fig. 10:**
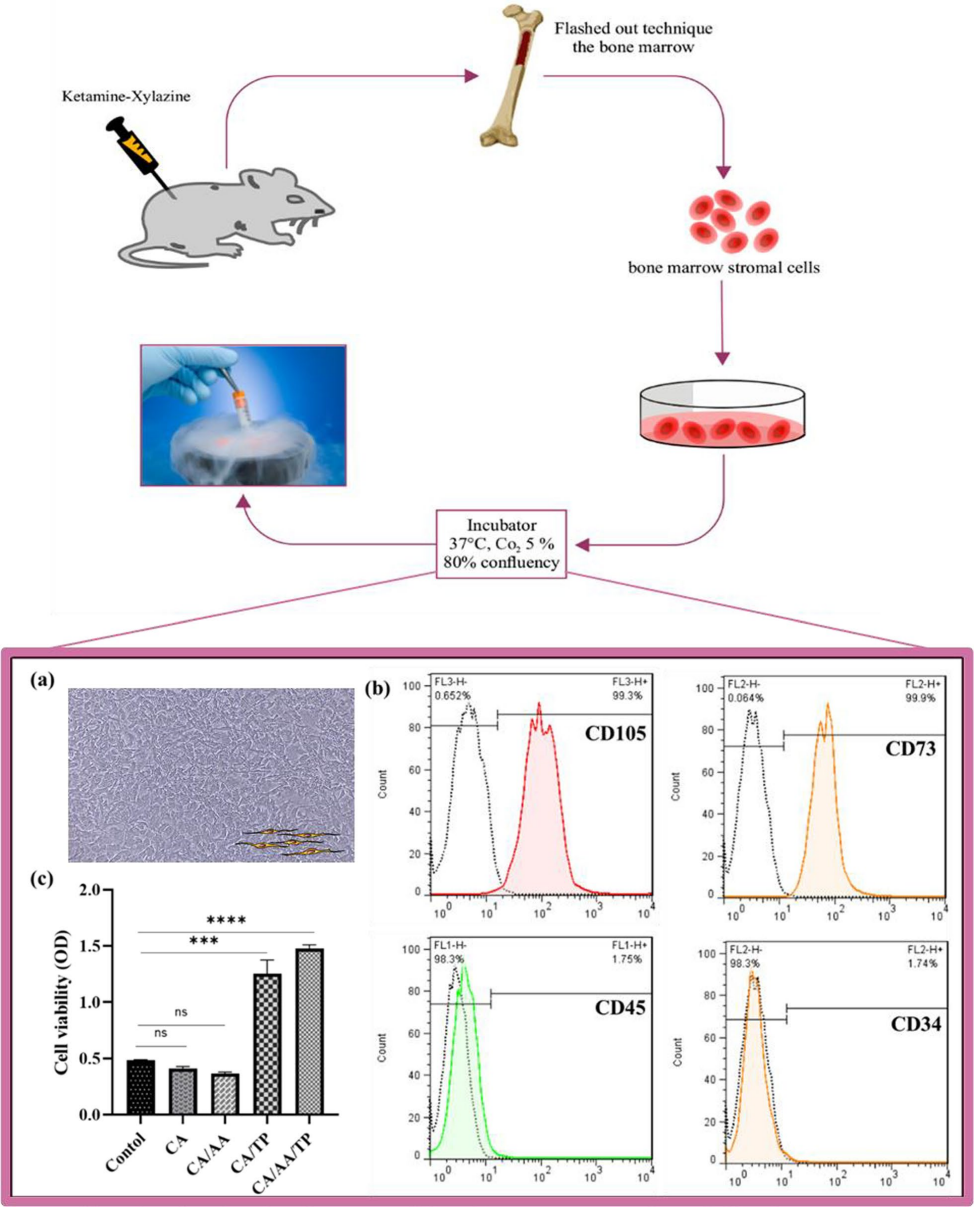
**a** Inverted light microscope image of rMSCs. MSCs are seen as attached and elongated. **b** Flow cytometry results illustrated that the expression of CD105 and CD73 surface markers was positive in rMSCs, while CD34 and CD45 surface markers was negative. **c** Histogram of the MTT assay after 72 h of cell seeding. Tow-Way ANOVA and Tukey post hoc analysis, ****p < .0001, ***p < .001, and “ns” represent no significant difference between groups

The inverted light microscope image (Fig. 10 a) showed the spindle-like shape of rMSCs cultured in the culture medium. FACS analysis was also used to identify surface markers of rMSCs. According to the findings shown in Fig. 10b, the extracted cells express the surface markers CD105 and CD75; however, they lack the hematopoietic markers CD34 and CD45.

MTT assay was investigated by seeding rMSCs on CA, CA/AA, CA/TP, and CA/AA/TP hydrogels at 72 h (Fig. 10c). According to MTT results, at 72 h after cell seeding, rMSCs viability in groups CA and CA/AA compared to the control group (plate wells without hydrogels) was not statistically significant, and it was slightly lower in CA and CA/AA. Additionally, the viability of rMSCs in CA/TP and CA/AA/TP groups was significantly higher than in other groups (*p* < 0.001).

**Fig. 11:**
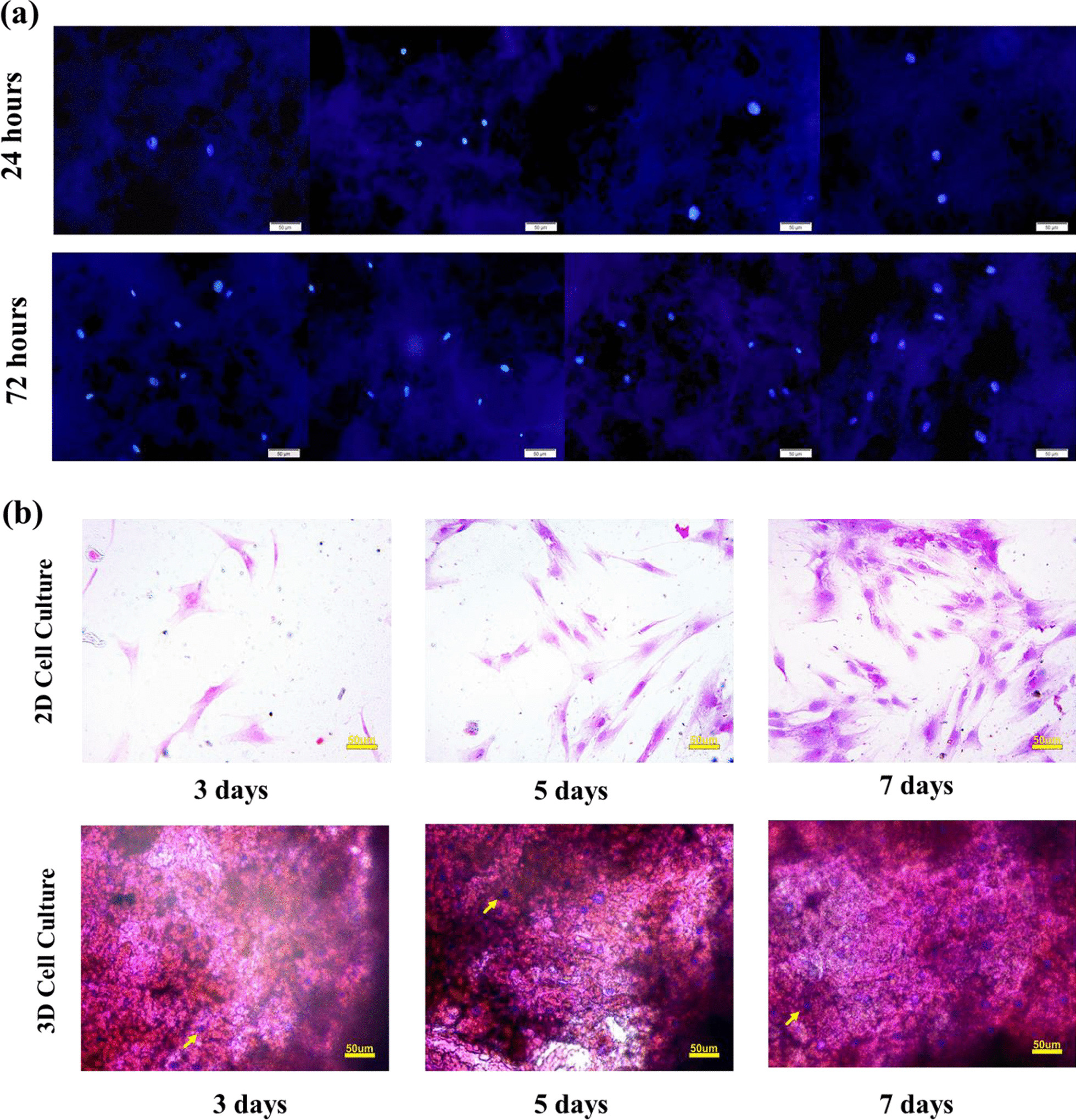
**a** DAPI nuclear staining on 24 and 72 h after cell culturing (magnification ×200), after 72 h of culturing, the number of cells increased about twice compared to 24 h. **b** H & E staining on 3, 5, and 7 days after 2D (magnification ×200) and 3D (magnification ×200) cell culturing. The purple color in the images indicates the presence of cell nuclei, as indicated by the yellow arrows. The number of cells on day seven after culturing has increased significantly compared to days 3 and 5, in both 2D and 3D cultures

DAPI nuclear staining was used to determine the cell viability and proliferation of rMSCs onto the CA hydrogel after 24 h and 72 h. The DAPI staining images (Fig. 11a) showed that 72 h after cell culture, cells were visible in nearly all the macropores of the hydrogels, and when compared to the first 24 h of culture, the number of nuclei has almost duplicated.

H&E staining was also used to demonstrate the presence and proliferation of cells inside the composite hydrogels after 3, 5, and 7 days of 2D and 3D cell culture. The H&E staining images (Fig. 11b) showed that 3, 5, and 7 days after cell culturing, the cells inside the hydrogel increased, and the most significant number of cells could be seen on day seven, and the pores in the composite hydrogels were filled with cells. These findings support the hypothesis that chitosan-alginate hydrogels promote cell proliferation and growth.

### Hypoxic assessments

**Fig. 12:**
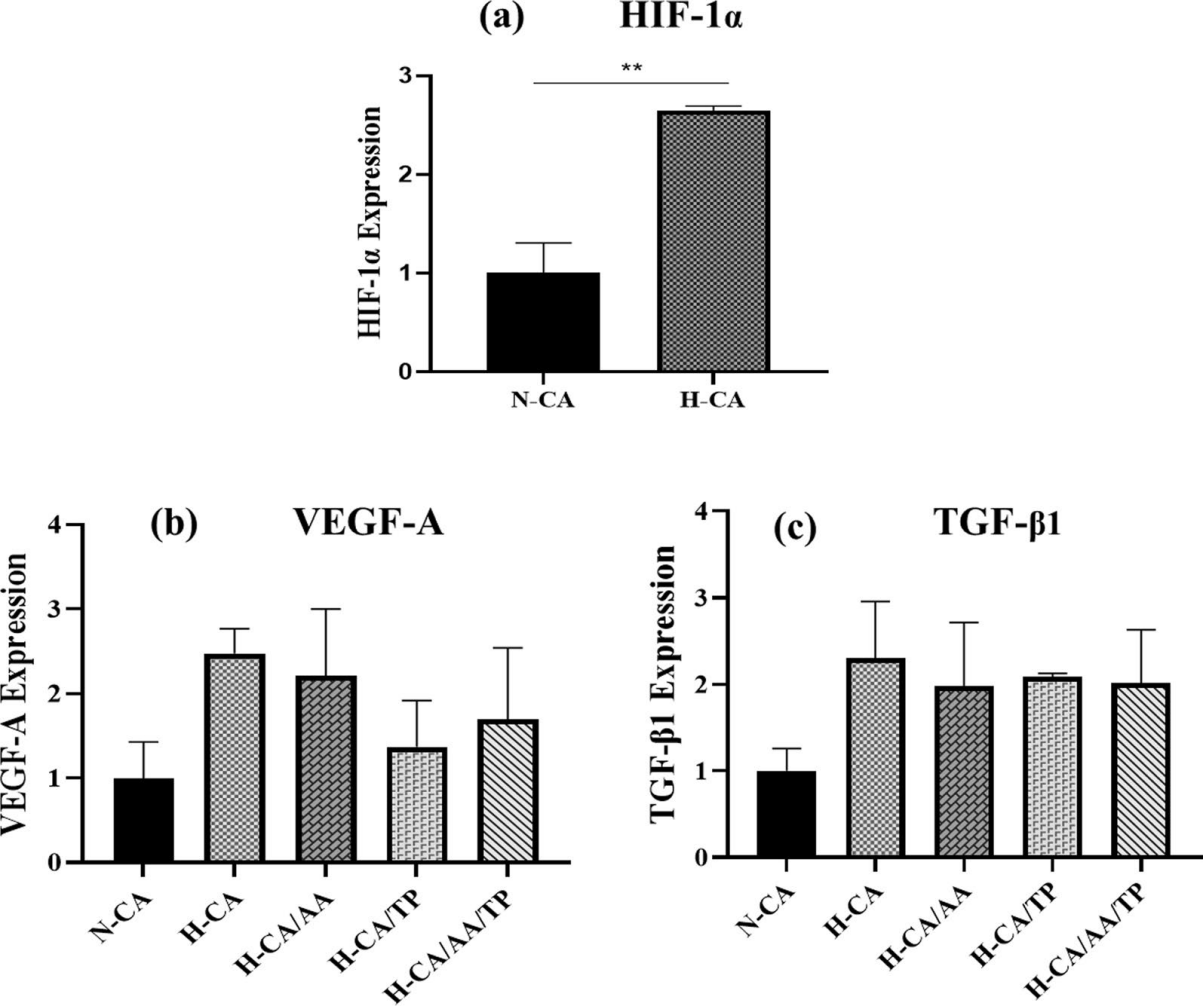
Real-time PCR analysis of HIF-1α, VEGF-A, and TGF-β1 (**a**–**c**, respectively) in normoxic and hypoxic conditions. **a** The HIF-1α expression level was significantly higher in the cells exposed to CoCl_2_ (H-CA group) compared to the N-CA group. **b** The expression level of VEGF-A was high in the H-CA group compared to other groups. In the presence of TP, compared to the H-CA, H-CA/AA, and H-CA/AA/TP groups, the expression of VEGF-A decreased. c) The expression of TGF-β1 had a high level of expression in all groups compared to the N-CA group, and in the H-CA group was higher than other groups (**p < *.01*)

Following seeding of rMSCs on CA, CA/AA, CA/TP, and CA/AA/TP composite hydrogels in normoxic and hypoxic conditions, real-time PCR analysis was conducted to examine the gene expression of HIF-1α to demonstrate the effect of CoCl_2_ as a hypoxia inducer (Fig. 12a) and to monitor the expression of known modulators involved in the wound-healing process, such as VEGF-A, TGF-β1 (Fig. 12b, c). The expression level of HIF-1α was significantly higher in the CoCl_2_-exposed cells (H-CA group) compared to the control group (N-CA), as shown by the graph in Fig. 12a. VEGF-A expression is depicted in Fig. 12b and showed that the expression level of VEGF-A rose in hypoxia as compared to normoxia. As can be seen, in the H-CA group compared to the N-CA group, the expression of the VEGF-A gene increased by about two times, and group H-CA displayed the highest profile of VEGF-A expression. The expression of VEGF-A in the H-CA/AA group was also high compared to the control, H-CA/TP, and H-CA/AA/TP groups. However, in the presence of TP, the expression of VEGF-A decreased compared to the H-CA, H-CA/AA, and H-CA/AA/TP groups, but it was still higher than the control group. The expression of TGF-β1 is depicted in Fig. 12c. As can be seen, compared to normoxia as a control group (N-CA), the expression of this gene significantly increased in the hypoxia groups. TGF-β1 expression is slightly higher in the H-TP group than in the other groups. These differences were not statistically significant.

## Discussion

Recently, researchers have concentrated on creating and developing efficient wound dressings that can aid in the quicker and more effective healing of wounds. One of the most effective wound dressings for accelerating the healing process is hydrogel-based dressings [44, 45]. Until today, many efforts have been made to make wound dressings or scaffolds suitable for skin tissue engineering to replace the skin or improve its regeneration. The structures prepared in this direction should be appropriate to the skin tissue in terms of physicochemical and biological properties. The purpose of this study was to examine the reparative properties of chitosan-alginate hydrogels cross-linked by CaCl_2_ and loaded with rMSCs cells in normoxic and hypoxic conditions, as well as specific dosages of antioxidants ascorbic acid and α-tocopherol in skin wound healing.

Comprehensively, in terms of porosity, weight loss, swelling, wettability, drug release, and hemolysis, the hydrogels investigated in this research presented excellent properties and were approved for use in skin tissue regeneration. Based on the SEM images, all composite hydrogels studied in this research are suitable for cell attachment and migration. According to studies, the optimal pore size which is suitable for skin tissue engineering is in the range of 100 to 200 μm, and these pores have an important role in cell migration and proliferation. When the vascular system does not work properly, nutrients and gas exchange occurs through these interconnected pores [46]. Also, scaffold degradation is another crucial aspect of tissue engineering and wound healing, because, with the gradual destruction of the scaffold, a favorable microenvironment is provided for the formation of new tissue [47]. In this regard, in the present study, CA/AA hydrogel showed the highest degradation rate compared to other groups due to high hydrophobicity.

Conversely, the application of hydrogels in skin tissue engineering is greatly influenced by their swelling behavior. Like the majority of natural polymers, alginate, and chitosan easily swell in biological fluids. The swelling rate analysis revealed that the composite hydrogels quickly absorbed PBS, and after two hours, reached a swelling equilibrium, and also, the CA and CA/AA groups, in comparison to other groups, became more swollen in 72 h. This demonstrates that the investigated composite hydrogels have the capacity to absorb exudate quickly and prevent exudate accumulation, thereby preventing infection at the wound site [2]. According to current researches, moist environments can facilitate wound healing. It implies that the capacity to retain moisture is crucial for tissue-engineered skin scaffolds [48]. On the other hand, the degree of hydrophilicity of the surface is a very effective factor in the capacity of cell attachment and proliferation [39]. Therefore, based on the contact angle results, all the studied samples were hydrophilic. Besides, we discovered that the hemolysis rate of the CA hydrogel in vitro, both with and without AA and TP, was below 5% and within the standard range. As a result, it was found that the amount of hemolysis caused by the addition of AA and TP does not exceed the permissible limit, so the designed wound dressings are highly compatible with blood. One of the crucial elements for the substances used in wound healing is hemolysis, which is defined as the release of hemoglobin into the plasma as a result of damage to RBC [2].

According to the research, CA hydrogel enriched with biologically active compounds, AA and TP, and loaded with hypoxic rMSCs may improve the overall effectiveness of the hydrogel by acting on several fronts. In vitro results of the MTT assays demonstrated that the addition of TP to CA hydrogels improved their suitability for cell proliferation due to their cyto-compatible nature [2]. Also, based on DAPI and H&E staining, these composite hydrogels successfully supported the attachment, growth, and proliferation of rMSCs, and the cells were visible in almost all macropores of the hydrogels. Therefore, it can be said that when CA hydrogels, especially CA/TP and CA/AA/TP hydrogels, are placed in the wound, fibroblasts and keratinocytes will penetrate the pores of the composite hydrogels, multiply and spread on them. Therefore, a suitable platform is provided for the interaction of cells with each other, which will allow the formation of a continuous layer, which is very important in restoring the integrity of the skin [49].

On the basis of the investigation, the wound-healing process requires the coordination of multiple cellular and molecular events, which are carried out by growth factors such as TGF-β1 and VEGF-A [22, 50, 51]. The current study showed that rMSCs expressed and secreted TGF-β-1 and VEGF-A under hypoxic conditions and in the presence of AA and TP. These growth factors have an impact on the migration, proliferation, and differentiation of resident cells and modify the regulation of the local environment [50]. Paracrine molecules derived from rMSCs have been considered a potential therapeutic strategy for wound healing because rMSCs have the potential to transdifferentiate into all three germ layers [50]. On the other hand, oxygen tension is one of the critical elements of the “niche” of stem cells and has recently been investigated and discussed as a factor in maintaining the ability of rMSCs to proliferate and paracrine mechanisms of regenerative function [50]. Based on this, the current study concentrated on the impact of AA and TP on the expression of TGF-β1 and VEGF-A in rMSCs as valuable components in the wound-healing process under hypoxic conditions. In the proliferative phase of wound healing, the role of AA is significant as it aids in the synthesis and maturation of collagen. It also plays a crucial role in the stabilization of collagen and is essential to the overall process of wound healing [52, 53]. Also, TP is renowned for its anti-inflammatory properties [54]. To maintain skin homeostasis in the unwounded epidermis, TGF-β1 inhibits the proliferation of keratinocytes and regulates their differentiation [55]. TGF-β1 plays a crucial role in all stages of wound healing by controlling the function of endothelial cells, fibroblasts, keratinocytes, and monocytes. TGF-β1 is quickly up-regulated and secreted by macrophages, monocytes, platelets, keratinocytes, and fibroblasts following acute injury. Moreover, TGF-β1 is a critical element in initiating inflammation and the formation of granulation tissue [55]. Furthermore, it promotes wound contraction by inducing the expression of α-smooth muscle actin (α-SMA) proteins in fibroblasts and inducing myofibroblast differentiation [55]. Subsequently, myofibroblasts, which are known as the critical drivers of progressive organ fibrosis, cause contraction of the wound edge and increase the production of extracellular matrix (ECM) components, including type I and III collagens, hyaluronan (HA), and fibronectin (FN) [56]. Various studies have shown that an exaggerated “wound repair” response mediated by TGF-β1 results in tissue thickening and scarring following excessive ECM deposition [57, 58]. In addition to stimulating keratinocyte migration, TGF-β1 also induces angiogenesis by upregulating VEGF [55]. According to real-time PCR data, the expression of the TGF-β1 gene increased in rMSCs under hypoxic conditions, and there was no significant difference in its increase between the groups containing AA and TP, so it can be concluded that HIF-1α was responsible for mediating the signaling pathway of TGF-β1. Based on the results, it appears that hypoxia contributes to up-regulation of TGF-β1, which plays a crucial role in the process of wound closure [51]. According to a study published in 2018 by Mingyuan et al. hypoxia promotes the activation of TGF-β1/Smad signaling through HIF-1α. This signaling, along with HIF-1α, contributes to collagen deposition under hypoxic conditions, which is an essential factor in the formation of keloids [59]. As a result, it can facilitate wound healing and ultimately leads to scar formation.

Multiple investigations have shown that VEGF plays a significant role in promoting neovascularization and angiogenesis during the healing process of wounds [60], and VEGF-A is a marker of inflammation and is one of the genes targeted by HIF-1α [54]. In this study, it was discovered that the expression level of the VEGF-A gene is controlled by the expression of the HIF1α gene. Mohammed et al. (2015) investigated the role of ascorbic acid in inflammatory phase of wound healing, proliferation, and maturation stages in mice. The researchers noticed that mice given enough ascorbic acid and supplemented with ascorbic acid showed higher levels of VEGF expression [60]. But there was no significant alteration in the expression of the VEGF-A gene in the presence of AA in our results. Conversely, in 2018, Bhatti et al. conducted a study to explore how vitamin E (TP) treatment affects H_2_O_2_-induced oxidative stress in porcine adipose-derived mesenchymal stem cells (p-ASCs). Compared to H2O2-induced p-ASCs, the treatment of vitamin E resulted in the down-regulation of VEGF-A gene expression levels [54]. In accordance with the study results, H2O2 increases the expression of HIF-1α, and HIF-1α cusses up-regulation of VEGF-A [54]. So, it can be stated that treatment of rMSCs with TP can potentially provide resistance against inflammation and oxidative stress.

Based on the data we have collected, hypoxia induces HIF-1α, TGF-β1, and VEGF-A expression levels, so it is predicted that this will increase collagen synthesis and promote wound contraction, accelerate the process of wound healing, and may resulting in scarring. Conversely, to improve the self-regeneration ability of the body, adding AA to the environment leads to a partial reversal of these changes. Therefore, it is known as an epigenetic regulator and can help reduce excessive scarring. Therefore, it can be concluded that AA can moderate the scar caused by hypoxia but currently, there are not enough data to confirm the pathways through which AA decreases HIF-1α activity levels, so on comprehensive researches is needed [61]. Our data also showed that TP leads to the reduction of VEGF-A gene expression and is expected to lead to the reduction of inflammation and oxidative stress, in order to gain further insight, it is necessary to conduct additional investigations.

## Conclusion

The current study examined the combined effects of CA hydrogels enriched by AA and TP on skin wound healing under normoxic and hypoxic conditions in vitro. Our findings demonstrated that the properties of the CA composite hydrogels are favorable for skin wound-healing applications and drug delivery due to the sustained release of the drug, and the pore size of CA composite hydrogels is suitable for cell adhesion and migration. In vitro*,* results of cell viability studies demonstrated that the addition of TP to CA hydrogels improved their suitability for cell proliferation. Another objective of the current study was to clarify and compare the effects of normoxic and hypoxic conditions on the expression and secretion of MSC-derived growth factors that theoretically contribute to cutaneous wound healing. Based on the findings of this research, the prepared composite hydrogels made of CA/AA/TP that were seeded by MSCs under hypoxic conditions may hold promise for effective wound healing by reducing the inflammation and oxidative stress at the wound site, increasing wound closure, and modulating scar formation.

## Data Availability

The data that support the findings of this study are available from the corresponding author upon reasonable request.

## Abbreviations

CA: Chitosan/alginate
AA: Ascorbic acid
TP: α-Tocopherol
CaCl_2_: Calcium chloride
SEM: Scanning electron microscope
FTIR: Fourier transform infrared
DAPI: 4′,6-Diamidino-2-phenylindole
H&E: Hematoxylin and eosin
3D: Three-dimensional
FDA: Food and Drug Administration
MRSA: Methicillin-resistant Staphylococcus aureus
PHDs: Prolyl hydroxylase domain proteins
MTT: 3-(4,5-Dimethylthiazol-2-yl)-2,5-diphenyltetrazolium bromide
rMSCs: Rat bone marrow mesenchymal stem cells
DMEM-LG: Low-Glucose Dulbecco’s Modified Eagle’s Medium
Pen/Strep: Penicillin–streptomycin
FBS: Fetal bovine serum
PBS: Phosphate-buffered saline
BM-SCs: Bone marrow mesenchymal stem cells
ELISA: Enzyme linked immunosorbent assay
RBC: Red blood cells
H: Hypoxia
N: Normoxia
α-SMA: α-Smooth muscle actin
ECM: Extracellular matrix
HA: Hyaluronan
FN: Fibronectin
pASCs: Adipose-derived mesenchymal stem cell
HIF-1α: Hypoxia-inducible factor-1α
TGF-β1: Transforming growth factor-β1
VEGF-A: Vascular endothelial growth factor A
CoCl_2_: Cobalt chloride
